# Structural Investigation of Beta-Cyclodextrin Complexes with Cannabidiol and Delta-9-Tetrahydrocannabinol in 1:1 and 2:1 Host-Guest Stoichiometry: Molecular Docking and Density Functional Calculations

**DOI:** 10.3390/ijms24021525

**Published:** 2023-01-12

**Authors:** Nat Triamchaisri, Pisanu Toochinda, Luckhana Lawtrakul

**Affiliations:** School of Bio-Chemical Engineering and Technology, Sirindhorn International Institute of Technology, Thammasat University, Pathum Thani 12120, Thailand

**Keywords:** complexation process, intermolecular interaction, Minnesota density M06-2X functional, structure-based molecular design

## Abstract

The complexation of β-cyclodextrin (β-CD) with cannabidiol (CBD) and Δ^9^-tetrahydrocannabinol (THC) was investigated using molecular docking and M062X/6-31G(d,p) calculations. The calculations suggested two possible complex formations of 1:1 and 2:1 host-guest molecular ratio of β-CD with CBD and THC. The preferred orientation of all complexes in this study exhibited the hydrogen bonding between hydroxy-substituted benzene ring of CBD and THC with the β-CD’s secondary hydroxy groups at the wide rim. The calculated complexation energies indicate that formation of the 2:1 complexes (−83.53 to −135.36 kcal/mol) was more energetically favorable and chemically stable than the 1:1 complexes (−30.00 to −34.92 kcal/mol). However, the deformation energies of the host and the guest components in the 2:1 complexes (37.47–96.91 kcal/mol) are much higher than those in the 1:1 complexes (3.49–8.69 kcal/mol), which means the formation processes of the 2:1 complexes are more difficult due to the rigidity of the dimeric β-CDs. Therefore, the inclusion complexes of β-CD with CBD and THC are more likely to be in 1:1 host-guest ratio than in 2:1 molecular ratio. The results of this study supported the experimental results that the complexation constant of 1:1 β-CD/CBD (Ks = 300 M^−1^) is greater than that of 2:1 β-CDs/CBD (Kss = 0.833 M^−1^). Altogether, this study introduced the fitting parameters that could indicate the stability of the molecular fits in complex formation of each stoichiometry host-guest ratio, which are important for the assessment of the inclusion mechanisms as well as the relationships of reactants and products in chemical reactions.

## 1. Introduction

Cannabidiol, often known as CBD, is a phytocannabinoid that is derived from the Cannabis plant. It does not produce any euphoric effects, but it does have pain-relieving [1], anti-inflammatory [2,3], anti-cancer [4], and cancer-preventing properties [5,6,7]. When CBD is taken orally, it immediately provides anti-proliferative, anti-angiogenic, and pro-apoptotic effects through a variety of mechanisms. Additionally, there is some evidence that CBD can encourage the uptake of cytotoxic chemicals by cancer cells. CBD is a cannabinoid that can be used as a treatment for children who suffer from refractory epilepsy as a result of Lennox-Gastaut syndrome or Dravet syndrome [8,9]. Δ^9^-tetrahydrocannabinol, also known as THC, is the principal component of the marijuana plant that is responsible for its euphoric and psychoactive effects. In 1964, Raphael Mechoulam, an Israeli chemist born in Bulgaria, made the initial discovery of THC and isolated it [10,11]. It was later discovered that when THC is smoked, it enters the circulation, travels to the brain, and attaches itself to naturally occurring cannabinoid receptors that are a part of the endocannabinoid system [12]. These receptors are found in the cerebral cortex, cerebellum, and basal ganglia [13,14]. These are the areas of the brain that are in charge of thinking, remembering, feeling pleasure, coordinating movement, and other functions [15,16]. However, the low water solubility of CBD (0.0126 mg/mL) [17,18,19,20] and THC (0.0028 mg/mL) [21] becomes an obstacle to their applications in pharmaceutical fields.

**Figure 1:**
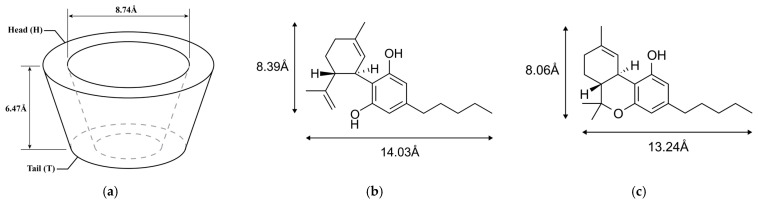
Schematic representation of the chemical structures and the dimensions of the energy minimized molecular conformation: (**a**) β-CD (C_42_H_70_O_35_) and its position of the wider rim (Head, H) and the narrow rim (Tail, T); (**b**) CBD (C_21_H_30_O_2_); (**c**) THC (C_21_H_30_O_2_).

In recent years, there have been several studies indicating that the water solubility and stability of these cannabinoids can be increased through the inclusion complex with β-cyclodextrins (β-CD), about 2.1 mg/mL [22,23,24,25,26]. These inclusion complexes also exhibit greater in vitro effects against human cancer lines Hep G2 and A549 [22,23]. β-CD is a macrocyclic ring consisting of seven glucose subunits joined by α-1, 4 glycosidic bonds. It has the shape of a truncated cone with a hydrophilic outer surface and lipophilic cavity [27]. All the secondary hydroxyl groups (corresponding to the C2 and C3 carbon atoms of the glucose units) are on the same edges of the cavity with wider rim (Head), whereas the primary hydroxyls are on the other end of the cavity with narrower rim (Tail). The cavity size is a major determinant as to which cyclodextrin (CD) should be used in complexation with hydrophobic molecules. The inner diameter of the wide rim of β-CD is 8.74 Å with a depth of 6.47 Å, as illustrated in Figure 1a. Several studies reported that CBD and THC can form complexes with β-CD in 1:1 and 2:1 host-guest stoichiometric ratios. The stoichiometry of the complex is given by the number of host and guest molecules contained in the inclusion complex. Lv et al. [22] reported that β-CD complexes with CBD in both of 1:1 and 2:1 host-guest stoichiometry. Li et al. [24] revealed that CBD formed inclusion complex with β-CD in 1:1 stoichiometric ratio. Mannila et.al. [25] achieved host-guest inclusion complex of randomly methylated β-CD with CBD and THC in 1:1 and 2:1 molecular ratio. Shoyama et al. [26] found a 2:1 stoichiometry in β-CD/THC system.

As the previous studies on the formation of β-CD complexes with CBD and THC implies the simultaneous presence of 1:1 and 2:1 complex, it is necessary to introduce fitting parameters to determine the reliability of the complex formation. Although many articles study the inclusion complexes of CBD and THC with CDs, as mentioned above, currently there are no articles investigating the molecular structures of β-CD/CBD and β-CD/THC in 1:1 and 2:1 host-guest stoichiometry, which are important for the assessment of the inclusion mechanisms as well as the relationships of reactants and products in chemical reactions. Therefore, in this work, molecular docking calculation was used to establish the most possible modes and calculate the binding energy of 1:1 and 2:1 complex formation of β-CD with CBD and THC. Furthermore, the energies of complexation, energy gap between HOMO (the highest occupied molecular orbital) and LUMO (the lowest unoccupied molecular orbital), and the deformation energies of the inclusion complex were assessed by density functional theory (DFT) calculations, to identify the fitting parameters that can indicate the stability of the molecular fits in complex formation of each stoichiometry host-guest ratio.

## 2. Results and Discussions

### 2.1. One-to-One Inclusion Complexes of β-CD with CBD and THC

**Figure 2:**
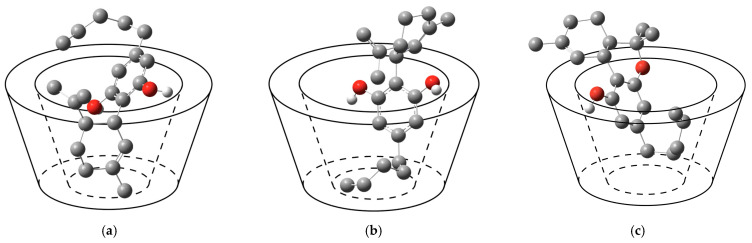
Schematic representations of the conformations of the 1:1 inclusion complex: (**a**) β-CD/CBD Conf. I (**b**) β-CD/CBD Conf. II; (**c**) β-CD/THC Conf. II.

The only difference between CBD and THC is a chemical bond (Figure 1b,c), which results in different structures, and chemical and pharmacological properties. Molecular docking is used to calculate the possibility of binding between β-CD either with CBD or THC molecules by fixing the host structure and allowing the guest to be flexible in the host’s cavity. The calculations revealed two possible orientations in the 1:1 host-guest stoichiometry case of β-CD/CBD and only one orientation for β-CD/THC, as shown in Table 1 and Figure 2.

In term of configurations, in conformation I (Conf. I), the limonene group of CBD is located inside the cavity closer to the narrower side of β-CD (Tail), while its aliphatic chain is elongated outside the cavity closer to the wider rim of β-CD (Head), as displayed in Figure 2a. In conformation II (Conf. II), the benzenediol group of CBD stayed inside the cavity near the wider rim, while its limonene group stayed outside the cavity closer to the wider rim, and its aliphatic chain elongated outside the cavity closer to the narrow rim of β-CD, as presented in Figure 2b. Molecular docking calculations indicated that β-CD/CBD in Conf. I (99% frequency; the lowest ΔG = −6.87 kcal/mol) was more favorable than Conf. II (1% frequency; ΔG = −6.07 kcal/mol). The calculation on β-CD/THC shows that the preferred configuration is Conf. II (100% frequency) with the lowest ΔG = −6.77 kcal/mol, as reported in Table 1. The complex’s orientation, which the aliphatic chain of guest molecule located closer to the narrow rim, would be referred to as Conf. II. Note that, in the β-CD/THC Conf. II, the aliphatic chain of THC was puckered inside the cavity, as depicted in Figure 2c, while in the β-CD/CBD Conf. II, the aliphatic chain of CBD is elongated outside the cavity of β-CD (Figure 2b).

**Figure 3:**
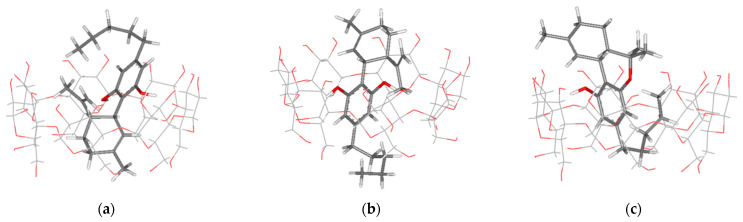
M062X/6-31g(d,p) energy-optimized structures of 1:1 inclusion complex: (**a**) β-CD/CBD Conf. I; (**b**) β-CD/CBD Conf. II; (**c**) β-CD/THC Conf. II.

Because the rigidity of β-CD molecule in molecular docking simulations was not realistic, the flexibility of the complex geometries and the intermolecular interactions of the 1:1 β-CD/CBD and β-CD/THC systems were further examined by M062X/6-31g(d,p) density functional calculations. The energy-optimized structures of the β-CD/CBD and β-CD/THC inclusion complexes are depicted in Figure 3. The M062X/6-31G(d,p) energy-optimized conformations of the inclusion complex systems demonstrated that the arrangements of guest molecules were related to the starting docking geometries, as presented in Figure 3. This indicated a good performance and reliability of the molecular docking techniques for this investigation.

Table 2 presents the distance of the intermolecular hydrogen bonds (H-bonds), which are found in M062X/6-31G(d,p) energy-optimized structures. In the studied complex systems, three types of H-bonds were identified. The first one, which is found in all systems, is between an ether-like anomeric oxygen atom in the *n* glucose unit of β-CD and a hydrogen atom of CBD’s hydroxyl group (O4_(β-CD)n_ ··· H_(OH-guest)_). The second one, which is found in β-CD/CBD Conf. I and β-CD/THC Conf. II, is from an oxygen atom of the secondary hydroxyl group at O2 of β-CD and the hydrogen atom of guest’s hydroxy group (O2_(β-CD)n+1_ ··· H_(OH-guest)_). The third one, which is found only in β-CD/THC Conf. II, occurs between the oxygen atom in benzo[c]chromene group of THC and the hydrogen atom of the secondary hydroxyl at O3 of β-CD (O_(THC)_ ··· H_(O3H-β-CD)n-2_).

The results obtained from M062X/6-31G(d,p) calculations for the 1:1 complexes are shown in Table 3, where µ_sp_ (component) is the dipole moment of the single-point energy of the guest or the host taken from the optimized complex. Dipole moment results from non-uniform distributions of positive and negative charges on the atoms inside the system. The dipole moment values of β-CD/CBD and β-CD/THC systems are closer to the dipole moment of free β-CD component than those of free CBD or THC, which means that the guest molecules are likely encapsulated inside the hydrophobic cavity of β-CD. The large |HOMO-LUMO| gap indicated that all inclusion complexes are as chemically stable as the free host and guest molecules. Complexation energy (ΔE) values are negative for all inclusion complexes which suggests that their formations are energetically favorable. Deformation energy $\left(E_{\mathrm{DEF}}\right)$ is the energy required to distort each monomer into the structure it adopts within the complex. The deformation energy of CBD molecule is higher than that of β-CD in both Conf. I and Conf. II, which indicates that the flexibility of the CBD structure plays an important role in stabilizing the whole system upon complexation. In the β-CD/THC complex, both components exhibit low value of deformation energies, which means that the complex formation is very favorable. This information is supported by small changes of µ_sp_ (component) values compared to the dipole moments of isolated guest and host molecules. According to the values of ΔE and $E_{\mathrm{DEF}}$, the stability of 1:1 inclusion complexes is in the following order: β-CD/THC Conf. II > β-CD/CBD Conf. II > β-CD/CBD Conf. I.

### 2.2. Two-to-One Inclusion Complexes of β-CDs with CBD and THC

**Figure 4:**
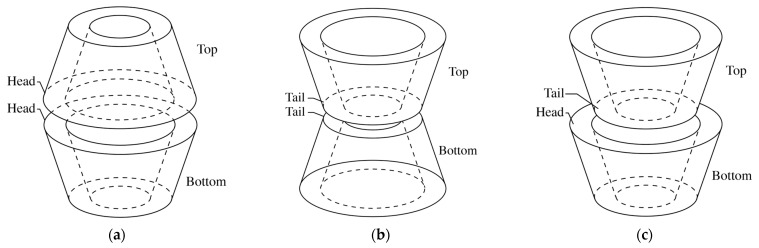
Schematic representation of the pairwise initial arrangement of β-CDs: (**a**) Head-to-Head (HH) configuration; (**b**) Tail-to-Tail (TT) configuration; (**c**) Tail-to-Head (TH) configuration.

In this section, 2:1 host-guest stoichiometry of β-CDs with CBD and THC inclusion complexes, wherein two β-CD molecules are fetched closer together, is investigated with three dimerized form of β-CDs: facing secondary portals (Head-to-Head, HH), facing two primary portals (Tail-to-Tail, TT), and facing primary and secondary portals (Tail-to-Head, TH), as shown in Figure 4a–c, respectively.

**Figure 5:**
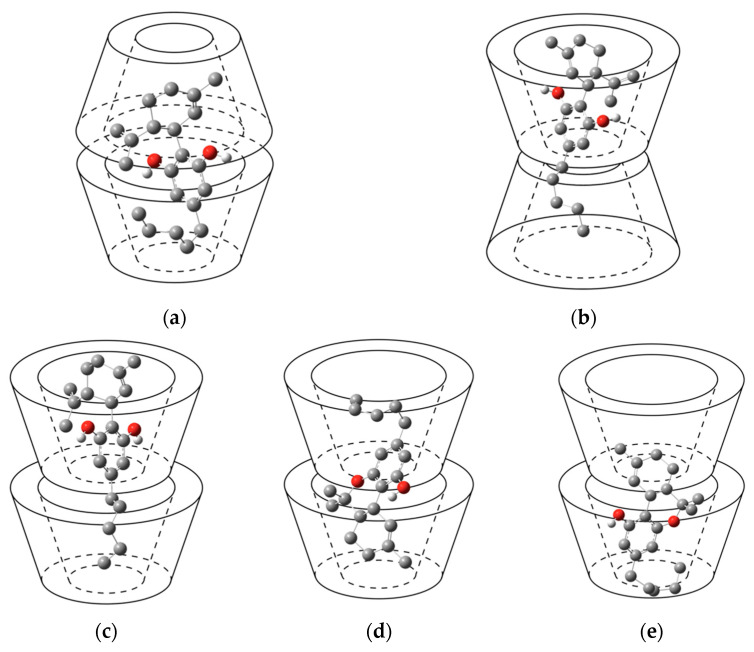
Schematic representation of five conformations of the 2:1 complex: (**a**) HH/guest Conf. III; (**b**) TT/guest Conf. IV; (**c**) TH/guest Conf. V; (**d**) TH/THC Conf. VI; (**e**) TH/THC Conf. VII.

Molecular docking calculation results in Table 4 indicate only one possible arrangement of CBD molecule inside HH, TT, and TH dimeric cavities, which hereafter would be referred to as Conformation III (Conf. III), Conformation IV (Conf. IV), and Conformation V (Conf. V), respectively. Figure 5 illustrates the orientations of CBD and THC inside the β-CDs dimer. The docking simulations of HH/THC and TT/THC complex systems also found only one preferred conformation, while in TH/THC system, three conformations were found, including Conf. V (80% frequency), Conformation VI (Conf. VI, 16% frequency), and Conformation VII (Conf. VII, 4% frequency), as presented in Figure 5. The binding energy (ΔG) of the 1:1 complexes is in the range of −6.07 to −6.87 kcal/mol (Table 1), while ΔG of the 2:1 complexes is in the range of −7.57 to −9.61 kcal/mol (Table 4), which demonstrates that the 2:1 host-guest ratio complexation is more energy favorable than the 1:1 host-guest ratio.

In terms of docking conformations, only one molecular arrangement was found in each of HH/CBD, TT/CBD, and TH/CBD complexes. In all of them, the cyclohexene group of CBD was located in β-CD_Top_, while its aliphatic chain is located in β-CD_Bottom_, as shown in Figure 5a–c. Due to its length, the benzenediol group of CBD was always found by the side of the wider rim of a β-CD molecule, when CBD is incorporated into the host cavity. Only one conformation of HH/THC and TT/THC inclusion complexes were obtained from molecular docking simulations, with similar molecular arrangement as β-CDs/CBD. However, in TH/THC system, the docking results obtain three different conformations, as depicted in Figure 5c–e. The first is TH/THC Conf. V, where THC was entrapped inside the dimeric cavity with its cyclohexene group located in the β-CD_Top_ and its aliphatic chain in β-CD_Bottom_ (Figure 5c), as previously observed in TH/CBD systems. On the other hand, in TH/THC Conf. VI, the cyclohexene group of THC was immersed deep into the narrow side of β-CD_Bottom_, and the hydroxy-substituted benzene ring located at the wider rim of the same β-CD_Bottom_, while its aliphatic chain settled in β-CD_Top_, as shown in Figure 5d. The third conformation, TH/THC Conf. VII, is analogous to 1:1 β-CD/THC Conf. II, where the aliphatic chain of THC molecule puckered inside the cavity with the hydroxy-substituted benzene ring locating at the wider rim of the same β-CD, as shown in Figure 5e.

**Figure 6:**
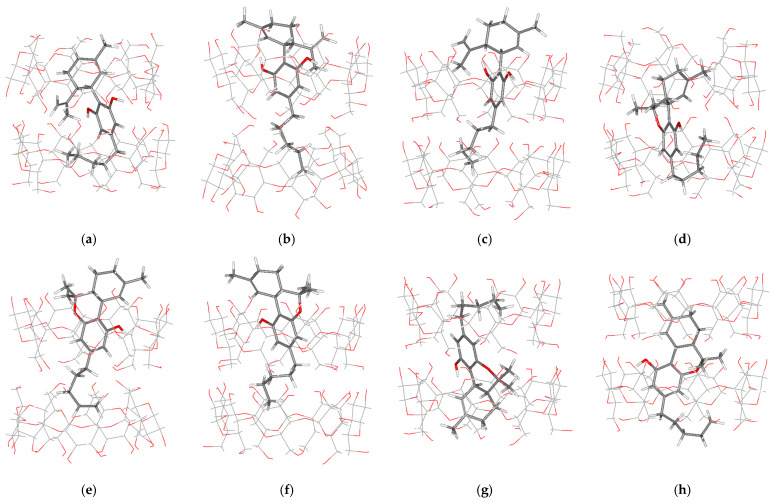
M062X/6-31g(d,p) energy-optimized structure of the 2:1 complex: (**a**) HH/CBD Conf. III; (**b**) TT/CBD Conf. IV; (**c**) TH/CBD Conf. V; (**d**) HH/THC Conf. III; (**e**) TT/THC Conf. IV; (**f**) TH/THC Conf. V; (**g**) TH/THC Conf. VI; (**h**) TH/THC Conf. VII.

Starting from the molecular docking geometry of each complex, the geometry was fully optimized without any constraint using M062X/6-31G(d,p) calculation. Figure 6 presents the energy-optimized conformations of 2:1 host-guest stoichiometry complex systems. The intermolecular H-bond distances between the host and the guest molecules, which are related to the complex stability, are reported in Table 5. Two types of H-bonds were found. The first type, which occurs in all complex systems, is between an ether-like anomeric oxygen atom in the *n* glucose unit of β-CD and a hydrogen atom of guest’s hydroxyl group (O4_(β-CD)n_ ··· H_(OH-guest)_). The second type, found only in HH/CBD Conf. III, is between the oxygen atom at O3 of β-CD and the hydrogen atom of the CBD’s hydroxyl group (O3_(β-CD)n_ ··· H_(O1H-CBD)_). Due to its size, the hydroxy-substituted benzene ring of CBD and THC is always located near the wider side of a β-CD molecule.

Table 6 presents the energy results from M062X/6-31G(d,p) calculations, expressed as complexation, HOMO and LUMO, and deformation energies. The formation of 2:1 host-guest ratio complexes is energetically favorable and chemically stable, as indicated by the negative complexation energy values (ΔE) and the large |HOMO-LUMO| gaps. The deformation of β-CDs is much stronger than the deformation of guest molecule. This is likely due to the rigidity of the dimeric β-CDs. The highest deformation energies for TH configurations (−88.7794 to −96.9119 kcal/mol, negative sign referring to a change in energy) means that complex formation should be difficult. TH and HH configurations exhibit more deformability upon complexation as suggested by higher deformation energy compared to TT configuration. Although the inclusion complexes with CBD and THC in TT configuration exhibit low deformation energy (−37.4730 and −38.3873 kcal/mol, respectively), they have unbalanced charge distribution as indicated by the high dipole moment (9.0612 debye and 8.4162 debye for TT/CBD and TT/THC, respectively), which ultimately lower the stability of compounds [28]. That is the reason why TT/guest systems have lower complexation energy than HH/guest and TH/guest systems. According to the values of ΔE, $E_{\mathrm{DEF}}$, and µ the orders of 2:1 inclusion complex stability is TH/guest Conf. V > HH/guest Conf. III > TT/guest Conf. IV.

Finally, as suggested by molecular docking and density functional calculations, both of 1:1 and 2:1 host-guest stoichiometry of β-CD with CBD and THC are feasible. The calculated complexation energies indicate that the formation of the 2:1 host-guest ratio complex is more energetically favorable and chemically stable than the 1:1 host-guest ratio complex. However, the deformation energies of the host and the guest components in the 2:1 complex are much higher than those in the 1:1 complex, which means the formation process of 2:1 is more difficult. This explains why the experimental complexation constant of 1:1 β-CD/CBD (*K*_s_ = 300 M^−1^) is greater than that of 2:1 β-CDs/CBD (*K*_ss_ = 0.833 M^−1^) [22].

## 3. Materials and Methods

### 3.1. Molecular Structure Construction

The starting geometries of β-CD, CBD, and THC monomers were taken from the Cambridge Crystallographic Data Centre (CCDC) with deposition number 1,107,192 [29], 1,533,487 [30], and 702,456 [31], respectively. The pairwise initial arrangement of β-CDs, with facing secondary portals (Head-to-Head, HH) and facing primary portals (Tail-to-Tail, TT), were extracted from CCDC 166671 [32] and CCDC 864,041 [33], respectively. Modification of each x-ray structure for atoms elimination and atoms addition was performed with Discovery Studio 2020 Client (DSC) [34]. Furthermore, DSC was also used to duplicate and rotate β-CD molecule to construct the dimeric β-CDs with facing primary and secondary portals (Tail-to-Head, TH), of which the X-ray structure was not available.

### 3.2. Molecular Docking Calculation

The inclusion complex between β-CDs with CBD and THC molecules were constructed by molecular docking calculations using AutoDockTools 1.5.7 and AutoDock 4.2 [35] software packages with the Lamarckian genetic algorithm [36]. The first step was the preparation of the host (β-CD) and the guests (CBD and THC) coordinate files using AutoDockTools. Non-polar hydrogens were deleted, and their charges were merged with carbon atoms. Atom types, hydrogen bond donors and acceptors, aliphatic, and aromatic carbon atoms, as well as rotatable bonds of the guest molecules, were also defined, while the hosts were kept fixed. AutoGrid was used to calculate the grid maps, one for each atom type present in the guest being docked—typically carbon, oxygen, and hydrogen atoms. The systems were investigated in a volume divided into many small grid boxes with a grid spacing of 0.375 Å, where the grid center was set at the center of the host molecules. The investigated systems were in the size of 19.5Å × 19.5Å × 19.5Å and 24.75Å × 24.75Å × 24.75Å for 1:1 and 2:1 host-guest stoichiometry complex systems, respectively. Finally, a hundred docking calculations were performed on each host-guest complex. At the end of docking simulations, the findings were grouped to identify related conformations, or clusters, based on the all-atom root mean square deviation within 2 Å. The binding energy (ΔG) and clustering information along with the coordinates for each docked conformation were recorded into the result files. The docked conformations with the lowest energy were selected for full geometry optimization, according to the previous studies [37,38,39,40].

### 3.3. Complexation Energy Calculation

Full geometry optimization was performed on the monomers as well as the 1:1 and 2:1 host-guest stoichiometry inclusion complex structures with the Minnesota density functional [41] M062X/6-31g(d,p) using the Gaussian 16 molecular modeling package [42]. The interaction between the host and the guest in the optimized geometries can be quantified by the complexation energy (ΔE) in Equation (1):

(1) $$\Delta {E=E}_{\mathrm{complex}}^{\mathrm{opt}}-(E_{\mathrm{host}}^{\mathrm{opt}}+E_{\mathrm{guest}}^{\mathrm{opt}})$$

where $E_{\mathrm{complex}}^{\mathrm{opt}}$, $E_{\mathrm{host}}^{\mathrm{opt}}$, and $E_{\mathrm{guest}}^{\mathrm{opt}}$ are the optimized energy of the inclusion complex, the isolated host molecule (β-CD), and the isolated guest molecule (CBD and THC), respectively. The lower complexation energy value, the more stable the inclusion complex. For the 2:1 host-guest stoichiometry inclusion complexes, the values of $E_{\mathrm{host}}$ were double the optimized energy of isolated β-CD. According to the DFT calculations in this study, an energy-optimized dimeric β-CDs structures without the guest was not obtained, as the β-CD molecules were separated from each other during the optimization. This behavior is supported by the information that aggregation of cyclodextrins can be promoted by the presence of guest compounds [43].

The deformation energy ($E_{\mathrm{DEF}}$) for each host and guest component throughout the formation of the inclusion complex was defined as in Equation (2):

(2) $$E_{\mathrm{DEF}}(\mathrm{component})=E{\left(\mathrm{component}\right)}_{\mathrm{sp}}^{\mathrm{opt}}\;-\;E{\left(\mathrm{component}\right)}^{\mathrm{opt}}$$

where $E{\left(\mathrm{component}\right)}_{\mathrm{sp}}^{\mathrm{opt}}$ is the single-point energy of the component taken from the optimized complex and $E{\left(\mathrm{component}\right)}^{\mathrm{opt}}$ is the energy of the optimized geometry of each free component.

## 4. Conclusions

In summary, we investigated the molecular structures of β-CD/CBD and β-CD/THC complexes in 1:1 and 2:1 host-guest stoichiometry. The calculated complexation energy and the HOMO and LUMO gaps of the inclusion complexes were used to indicate the stability of the molecular fits of the complexes’ formation with each stoichiometry host-guest ratio, which demonstrate that the 2:1 host-guest ratio complexation is more energy favorable than the 1:1 host-guest ratio. According to the values of ΔE, $E_{\mathrm{DEF}}$, and µ, the stability of 2:1 inclusion complex is in the following order: TH/guest Conf. V > HH/guest Conf. III > TT/guest Conf. IV. However, the deformation energies of dimeric β-CDs in 2:1 ratio were significantly higher than those of β-CD molecule in 1:1 ratio, which means the formation process with dimeric β-CDs is more difficult. Therefore, the inclusion complex of β-CD with CBD and THC is more likely to be in a 1:1 host-guest ratio than in a 2:1 molecular ratio.

## Figures and Tables

**Table 1:** The lowest and the average values of free energy of binding (ΔG) of β-CD/CBD and β-CD/THC inclusion complexes (1:1 host-guest ratio), and the frequency of conformations in a cluster obtained from molecular docking calculations at 298.15 K.

Host/Guest	Cluster	Conformation	Frequency (%)	ΔG (kcal/mol)	
				Lowest	Average
β-CD/CBD	1	I	41	−6.87	−6.57
	2	I	46	−6.81	−6.62
	3	I	12	−6.73	−6.49
	4	II	1	−6.07	−6.07
β-CD/THC	1	II	44	−6.77	−6.73
	2	II	26	−6.72	−6.64
	3	II	25	−6.71	−6.68
	4	II	4	−6.41	−6.35

**Table 2:** Distance of hydrogen bonds between host (β-CD) and guest (CBD and THC), obtained from M062X/6-31g(d,p) energy-optimized structures.

Inclusion Complex		Distance (Å)
β-CD/CBD Conf. I	O4_(β-CD)n_ ··· H_(O1H-CBD)_	1.84
	O2_(β-CD)n+1_ ··· H_(O3H-CBD)_	1.99
	O4_(β-CD)n+2_ ··· H_(O3H-CBD)_	2.50
β-CD/CBD Conf. II	O4_(β-CD)n_ ··· H_(O1H-CBD)_	1.90
	O4_(β-CD)n+3_ ··· H_(O3H-CBD)_	1.92
β-CD/THC Conf. II	O4_(β-CD)n_ ··· H_(O1H-THC)_	2.04
	O2_(β-CD)n+1_ ··· H_(O1H-THC)_	2.24
	O_(THC)_ ··· H_(O3H-β-CD)n-2_	2.85

**Table 3:** Complexation, deformation, HOMO and LUMO energies and dipole moment (µ) of 1:1 inclusion complex of β-CD/CBD and β-CD/THC calculated by M062X/6-31g(d,p) method.

	β-CD	CBD	THC	β-CD/CBD Conf. I	β-CD/CBD Conf. II	β-CD/THC Conf. II
µ (debye)	3.5332	1.7515	1.0617	4.7651	3.5766	3.1873
µ_sp_ (guest) (debye)				3.0744	3.3216	1.0512
µ_sp_ (host) (debye)				3.2881	3.4526	3.5170
HOMO (eV)	−8.7310	−6.9408	−6.9302	−7.2456	−7.1514	−7.1133
LUMO (eV)	2.2550	1.2640	1.2583	0.9625	0.8041	0.8041
Δ|HOMO-LUMO| (eV)	10.9860	8.2048	8.1884	8.2080	7.9555	7.9174
Energy (hartree) ^a^	−4273.8373	−968.3414	−968.3654	−5242.2266	−5242.2302	−5242.2583
ΔE (kcal/mol)				−29.9964	−32.3017	−34.9201
$E_{\mathrm{DEF}}\left(\mathrm{guest}\right)$ (kcal/mol)				12.0319	11.6836	2.9349
$E_{\mathrm{DEF}}\left(\mathrm{host}\right)$ (kcal/mol)				8.6929	4.7126	3.4940

^a^ 1 hartree = 627.5095 kcal/mol.

**Table 4:** The lowest and the average values of free energy of binding (ΔG) of dimeric β-CDs/CBD and dimeric β-CDs/THC inclusion complexes (2:1 host-guest ratio), and the frequency of conformations in a cluster obtained from molecular docking calculations at 298.15 K.

Host/Guest	Cluster	Conformation	Frequency (%)	ΔG (kcal/mol)	
				Lowest	Average
HH/CBD	1	III	55	−8.97	−8.63
	2	III	16	−8.69	−8.48
	3	III	14	−8.66	−8.50
	4	III	10	−8.64	−8.44
	5	III	5	−8.61	−8.42
TT/CBD	1	IV	38	−8.98	−8.72
	2	IV	61	−8.52	−8.12
	3	IV	1	−7.82	−7.82
TH/CBD	1	V	91	−7.74	−7.50
	2	V	5	−7.65	−7.40
	3	V	4	−7.57	−7.38
HH/THC	1	III	51	−9.61	−9.51
	2	III	43	−9.49	−9.41
	3	III	6	−9.12	−9.04
TT/THC	1	IV	59	−8.77	−8.70
	2	IV	37	−8.67	−8.58
	3	IV	4	−8.23	−8.18
TH/THC	1	V	41	−8.32	−8.12
	2	VI	16	−8.30	−8.20
	3	VII	4	−8.22	−8.15
	4	V	33	−8.11	−8.02
	5	V	6	−8.09	−8.03

**Table 5:** Distances of hydrogen bonds between the host (dimeric β-CDs) and the guest molecules (CBD and THC) obtained from the M062X/6-31g(d,p) energy-optimized structures.

Inclusion Complex		Distance (Å)
HH/CBD Conf. III	O3_(β-CD-Top)n_ ··· H_(O1H-CBD)_	1.99
	O4_(β-CD-Top)n_ ··· H_(O1H-CBD)_	3.10
TT/CBD Conf. IV	O4_(β-CD-Top)n_ ··· H_(O3H-CBD)_	1.86
TH/CBD Conf. V	O4_(β -CD-Top)n_ ··· H_(O1H-CBD)_	1.82
HH/THC Conf. III	O4_(β -CD-Bottom)n_ ··· H_(O1H-THC)_	2.78
TT/THC Conf. IV	O4_(β -CD-Top)n_ ··· H_(O1H-THC)_	2.08
TH/THC Conf. V	O4_(β -CD-Top)n_ ··· H_(O1H-THC)_	1.92
TH/THC Conf. VI	O4_(β -CD-Bottom)n_ ··· H_(O1H-THC)_	2.42
TH/THC Conf. VII	O4_(β -CD-Bottom)n_ ··· H_(O1H-THC)_	2.22

**Table 6:** Complexation, deformation, HOMO and LUMO energies, and dipole moment (µ) of 2:1 complexes of β-CD/CBD and β-CD/THC calculated by M062X/6-31g(d,p) method.

	HH/CBD Conf. III	TT/CBD Conf. IV	TH/CBD Conf. V	HH/THC Conf. III	TT/THC Conf. IV	TH/THC Conf. V	TH/THC Conf. VI	TH/THC Conf. VII
µ (debye)	4.9402	9.0612	3.8115	1.8504	8.4162	1.7925	2.2440	5.8098
µ_sp_ (guest) (debye)	2.4524	0.9196	2.6002	1.0526	1.0390	1.0210	3.0658	2.0765
µ_sp_ (host) (debye)	2.6626	9.3617	2.7540	3.0119	9.4903	2.5470	3.8716	3.4883
HOMO (eV)	−7.3291	−7.1884	−7.4964	−7.4328	−7.1522	−7.3013	−7.6812	−7.6439
LUMO (eV)	0.6705	0.8346	0.4991	0.4484	0.9094	0.6318	0.2392	0.2963
Δ|HOMO-LUMO| (eV)	7.9996	8.0230	7.9955	7.8812	8.0616	7.9332	7.9204	7.9403
Energy (hartree) ^a^	−9516.2118	−9516.1492	−9516.2214	−9516.2239	−9516.1777	−9516.2558	−9516.2402	−9516.2290
ΔE (kcal/mol)	−122.7734	−83.5332	−128.7802	−115.3329	−86.3642	−135.3628	−125.5592	−118.542
$E_{\mathrm{DEF}}\left(\mathrm{guest}\right)$ (kcal/mol)	10.0163	8.2348	8.9426	7.4027	4.7377	2.2892	8.8366	12.7880
$E_{\mathrm{DEF}}\left(\mathrm{host}\right)$ (kcal/mol)	−70.2202	−37.4730	−95.2610	−72.5865	−38.3873	−96.9119	−88.7794	−87.8827

^a^ 1 hartree = 627.5095 kcal/mol.

## Data Availability

The data presented in the study are included in the article. Further inquiries can be directed to the corresponding author.
